# Sorafenib Versus Apatinib Both Combined Transarterial Chemoembolization for Hepatocellular Carcinoma With Portal Vein Tumor Thrombosis: A Comparative Retrospective Study

**DOI:** 10.3389/fonc.2021.673378

**Published:** 2021-08-03

**Authors:** Yanyan Cao, Tao Sun, Xiaopeng Guo, Tao Ouyang, Xuefeng Kan, Lei Chen, Bin Liang, Mingfu Wang, Chuansheng Zheng

**Affiliations:** ^1^Department of Radiology, Union Hospital, Tongji Medical College, Huazhong University of Science and Technology, Wuhan, China; ^2^Hubei Province Key Laboratory of Molecular Imaging, Wuhan, China; ^3^Department of Radiology, The Third People’s Hospital of Hubei Province, Wuhan, China

**Keywords:** hepatocellular carcinoma, portal vein tumor thrombosis, sorafenib, apatinib, transarterial chemoembolization

## Abstract

**Objective:**

To compare the efficacy and safety of transarterial chemoembolization (TACE) combining with sorafenib or apatinib for hepatocellular carcinoma (HCC) patients with portal vein tumor thrombosis (PVTT).

**Methods:**

From June 2015 to March 2020, a total of 89 consecutive advanced HCC patients with PVTT who were treated with sorafenib-TACE (S-TACE) or apatinib-TACE (A-TACE) in our center were enrolled. The overall survival (OS), time to progression (TTP), tumor response, and adverse events in the two groups were compared.

**Results:**

There were 32 and 41 patients included in the S-TACE group and A-TACE group, respectively. The median follow-up was 10.0 months (range, 3.0–36.0 months) in the whole study. The median OS (11.0 *vs.* 10.0 months, *P* = 0.419), median TTP (5.0 *vs.* 6.0 months, *P* = 0.073), and tumor response (*P* = 0.529) between the S-TACE group and the A-TACE group were not significantly different. The adverse events related to sorafenib or apatinib were tolerable.

**Conclusion:**

S-TACE and A-TACE exhibited comparable prognosis for HCC patients with PVTT, which provide another effective and safe method of A-TACE for these patients except for conventional S-TACE.

## Introduction

Hepatocellular carcinoma (HCC), one of the most common malignancies, is the fourth leading cause of cancer-related death worldwide (1). The mortality rates of HCC ranks the third among various cancers in China (2). Although the mechanism is still poorly understood, HCC often invades the portal venous system, with the incidence rate of portal vein tumor thrombosis (PVTT) up to 10–60% (3). PVTT is one of the significant risk factors for the poor prognosis of HCC, and is an important leading cause of HCC-related death in Barcelona Clinic Liver Cancer (BCLC) stage C patients, with a median overall survival (OS) range of 2.7–4.0 months in non-treated people (4, 5).

As a multitargeted tyrosine-kinase inhibitor of the Raf and the vascular endothelial growth factor receptor-2 (VEGFR-2), sorafenib has been approved by FDA in 2007 and recommended as the first-line therapy for advanced HCC by the BCLC guideline. Clinical trials of comparing the efficacy of targeted drugs like sunitinib, brivanib, linifanib, and erlotinib to that of sorafenib fail to achieve a superiority or non-inferiority as first-line therapies for HCC patients (6–9). Although sorafenib is also able to improve the prognosis of HCC patients with PVTT (10, 11), it is not routinely recommended for HCC patients in clinical practice in China because of its high medical cost but modest survival benefits.

Transarterial chemoembolization (TACE), infusing chemotherapy agents and embolizing tumor feeding arteries through catheter, is the most common treatment for BCLC stage C HCC according to the BRIDGE study involving 18031 patients from 14 countries worldwide (12). Systemic reviews and meta-analyses demonstrated that HCC patients can comparably benefit from TACE to transarterial radioembolization, and transarterial radioembolization achieves a similar OS compared with sorafenib in the treatment of unresectable HCC, further indirectly proving the efficacy of TACE (13, 14). What is more, several researches have demonstrated in recent years that the combination of TACE and sorafenib could achieve a better prognosis of HCC patients with PVTT when compared with either TACE or sorafenib alone (15, 16).

Apatinib, a novel tyrosine kinase inhibitor, selectively targets VEGFR-2 with a binding affinity 10-fold that of sorafenib. In China, apatinib has been approved recently for the treatment of HCC due to its satisfactory efficiency. A randomized clinical trial showed that apatinib prolongs the OS of BCLC stage B or C HCC patients, which has also been validated in several retrospective studies (17–20). In addition, several researches indicated that apatinib is effective in advanced HCC patients with PVTT when combined with TACE (21, 22).

Although TACE combining with either sorafenib (S-TACE) or apatinib (A-TACE) could improve the outcome of HCC patients with PVTT, a comparison study of them is absent. Thus, the present study was designed to directly assess the efficacy of S-TACE and A-TACE.

## Materials and Methods

### Patients

The study was approved by the Ethics Committee of Tongji Medical College, Huazhong University of Science and Technology, and was conducted according to the Declaration of Helsinki. Informed consent was waived for the retrospective type of this study. The information of all participants is maintained with confidentiality.

From June 2015 to March 2020, a total of 89 consecutive HCC patients with PVTT treated with S-TACE or A-TACE in our center were enrolled.

Inclusion criteria: (1) age of 18–75 years; (2) diagnosed with HCC according to the American Association for the study of Liver Disease guidelines or European Association for the study of Liver; (3) PVTT confirmed by contrast-enhanced CT or MRI before the TACE procedure; (4) Eastern Cooperative Oncology Group (ECOG) score ≤2 points; (5) Child-Pugh A or B liver function; (6) platelet count ≥60 × 10^9^/L, neutrophil count >1.5 × 10^9^/L, hemoglobin >9 g/dl, prothrombin time <6 s above the upper limit of normal; (7) aspartate aminotransferase and alanine aminotransferase <200 U/L and total bilirubin ≤50 μmol/L.

Exclusion criteria: (1) with serious comorbidities, such as severe dysfunction of the kidney, lung, heart, or decompensated liver disease (ascites not controlled with diuretics, encephalopathy, active or recent [2 weeks] gastrointestinal bleeding); (2) previous history of liver resection, systemic chemotherapy, TACE, or other local-regional therapies; (3) other treatments during this period, such as iodine 125 seed implantation, radiofrequency ablation, external beam radiotherapy, or percutaneous ethanol injection; (4) other malignant tumors in addition to HCC.

### Sorafenib and Apatinib Administration

Sorafenib 400 mg was orally taken twice daily, and apatinib 500 mg/day was orally administrated initially, 3–5 days after each TACE procedure. The dose of sorafenib and apatinib was adjusted 1–2 weeks after the initial administration according to the individualized tolerance. The initial dose was maintained if patients could be well tolerant or have mild adverse events. If adverse events with equal to or greater than grade 3 defined by the National Cancer Institute Common Terminology Criteria for Adverse Events (version 4.0), the dose of sorafenib was reduced to 400 mg/day or 200 mg twice daily, and the dose of apatinib was reduced to 250 mg/day. If patients were still unable to be tolerant, the drug administration was temporarily interrupted or interrupted periodically once or twice every week. After a close observation for 1–2 weeks, drug administration could be gradually recovered in those with degraded or eliminated adverse events. Patients who had an interruption for more than 1 month were excluded from the study.

### TACE Procedure

The TACE procedure was conducted by operators who had at least 10-year experiences in interventional therapy. The Seldinger method was adopted to introduce a 5 French catheter (Cook, Bloomington, IN, USA) or combined with a 3 French microcatheter (Progreat, Terumo, Tokyo, Japan) into the tumor feeding arteries. Then, the emulsion was prepared by mixing 10–20 ml of lipiodol (Lipiodol Ultrafluido, Guerbet, France) with 20–40 mg doxorubicin hydrochloride (Hisun Pharmaceutical Co. Ltd., Zhejiang, China). The emulsion was injected into tumor feeding arteries through the microcatheter, followed by supplement embolization with gelatin sponge particles (300–700 µm, Cook Medical, Bloomington, IN, USA) until the stagnation of artery flow appeared.

### Follow-Up and Repeated TACE

All patients were regularly followed-up by scheduled protocols. The first follow-up was conducted at 4 weeks after the first TACE procedure. The next follow-up interval was extended to every 2 or 3 months. All patients were examined by adverse events through a thorough inquiry, laboratory tests, and physical examinations. Laboratory data included proteinuria, prothrombin time, α-fetoprotein (AFP), total bilirubin, serum albumin, thyroid-stimulating hormone, triiodothyronine (T3), thyroxine (T4), and free T4. Physical examination included abdominal contrast-enhanced computed tomography (CT) or contrast-enhanced magnetic resonance (MR). When an intrahepatic recurrent tumor or residual viable tumor was revealed on CT or MR images, a repeated TACE was performed in patients with Child-Pugh class A or B, and compensatory hepatic function (e.g., without uncontrolled ascites or hepatic encephalopathy).

### Assessments

The clinical, laboratory, and radiologic records of recruited patients were reviewed. Sorafenib- and apatinib-related adverse events were monitored until drug discontinuation. TACE-related adverse events that occurred within 1 month after the operation were evaluated in the two groups. Total bilirubin level, albumin level, and prothrombin time were documented 4 weeks after the first TACE procedure to evaluate the liver toxicity in the two groups. Postembolization syndrome, including abdominal pain, fever without infection, nausea, and vomiting, was expected and therefore not recorded separately.

Tumor response was evaluated according to the modified Response Evaluation Criteria or mRECIST, in solid tumors (23), by an experienced radiologist who was blinded to the survival data and treatment information. Time to progression (TTP) was defined as the time from the start of the first S-TACE or A-TACE session to the date that tumor progression was confirmed radiologically. OS was defined as the duration from the first S-TACE or A-TACE procedure to the death or the last follow-up.

The type of portal vein tumor thrombus (PVTT) was defined according to Cheng’s classification (24) as follows: Type I, tumor thrombus involving segmental or sectoral branches of the portal vein or above; Type II, tumor thrombus involving the right/left portal vein; Type III, tumor thrombus involving the main portal vein; and Type IV, tumor thrombus involving the superior mesenteric vein.

### Statistical Analysis

Continuous variables were summarized as mean ± standard deviation and analyzed by the independent samples t-test or the Mann-Whitney U-test. Qualitative variables were compared by the Chi-square test. The OS and the TTP curves were obtained using the Kaplan-Meier method and compared by the log-rank test. Cox proportional hazards model was used to calculate the hazard ratio (HR) with 95% confidence intervals (CI). All the analyses were performed using the SPSS version 25.0 software (IBM, Armonk, NY, USA). All statistical tests were two-tailed. *P* < 0.05 was considered statistically significant.

## Results

### Baseline Characteristics

**Figure 1:**
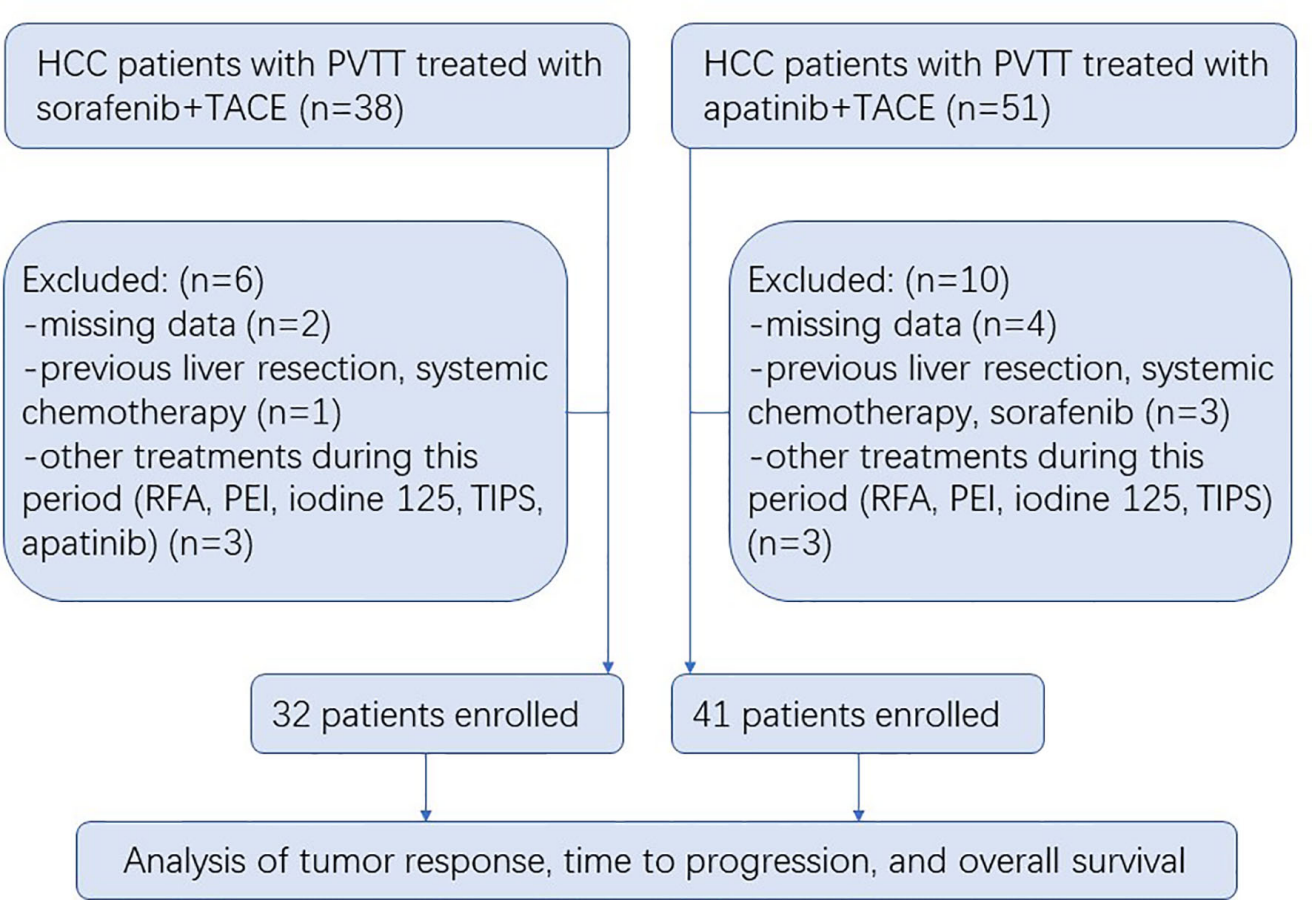
Flow diagram showing the patient selection process. HCC, hepatocellular carcinoma; PVTT, portal vein tumor thrombosis; TACE, transarterial chemoembolization; RFA, radiofrequency ablation; PEI, percutaneous ethanol injection; TIPS, transjugular intrahepatic portosystemic shunt.

A total of 89 HCC patients with PVTT received either S-TACE (n = 38) or A-TACE (n = 51) in the study. There were 6 and 10 excluded cases in S-TACE and A-TACE group, respectively, due to missing data, previous treatment, or other treatments during this period (**Figure 1**). No significant differences in baseline characteristics, including the age, gender, Child-Pugh class, liver functions, AFP, ECOG, HBV infection, tumor number and diameter, PVTT classification, ascites, extrahepatic spread, and TACE number, were identified between groups (**Table 1**).

**Table 1:** Baseline characteristics of patients with HCC and PVTT in the S-TACE or the A-TACE group.

Characteristics	Sorafenib + TACE group (n = 32)	Apatinib + TACE group (n = 41)	*P* value
Age (years)	52.7 ± 12.4	51.6 ± 9.6	0.678
Gender			0.723
Male	28	37	
Female	4	4	
Child-Pugh class			0.544
A	23	32	
B	9	9	
Bilirubin (μmol/l)*	19.00 (13.90, 30.30)^#^	16.10 (12.35, 21.95)^#^	0.397
Albumin (g/l)	34.98 ± 5.22	36.65 ± 5.07	0.173
PT (s)	13.77 ± 1.01	14.24 ± 1.78	0.180
AFP level (ng/ml)			0.644
<400	15	17	
≥400	17	24	
ECOG			0.746
0	21	34	
1	11	8	
HBV infection			0.406
Yes	24	37	
No	8	4	
Number of tumors			0.156
≤3	24	36	
>3	8	5	
Tumor diameter (cm)	4	14	0.054
≤5	28	27	
>5	8.40 (6.43, 12.00)^#^	7.20 (3.70, 11.15)^#^	
median*			0.128
PVTT type			0.522
1	10	13	
2	21	24	
3	1	4	
Ascites			0.081
Present	18	31	
Absent	14	10	
Extrahepatic spread			0.845
Present	18	24	
Absent	14	17	
TACE No.	2.9 ± 1.6	3.7 ± 1.9	0.065

*Analyzed with the Mann-Whitney U-test.

^#^Represents median (interquartile range).

HCC, hepatocellular carcinoma; PVTT, portal vein tumor thrombosis; S-TACE, sorafenib-transarterial chemoembolization; A-TACE, apatinib-transarterial chemoembolization; AFP, a-fetoprotein; ECOG, Eastern Cooperative Oncology Group.

### Treatment Efficacy

There were 1, 10, 10, and 11 patients in the S-TACE group that had complete response (CR), partial response (PR), stable disease (SD), and progressive disease (PD), respectively. The objective response rate (ORR) and disease control rate (DCR) in the S-TACE group were 34.4% and 65.6%, respectively. There were 1, 19, 6, and 15 patients in the A-TACE group that had CR, PR, SD, and PD, respectively. The ORR and the DCR were 48.7% and 63.4%, respectively. There was no significant difference between the two groups (*P* = 0.529) (**Table 2**).

**Table 2:** Tumor responses for patients with HCC and PVTT in the S-TACE or the A-TACE group.

Response	Sorafenib + TACE group (n = 32) (cases %)	Apatinib + TACE group (n = 41) (cases %)	*P* value
Complete response	1 (3.1)	1 (2.4)	0.529
Partial response	10 (31.3)	19 (46.3)	
Stable disease	10 (31.3)	6 (46.3)	
Progressive disease	11 (34.4)	15 (36.6)	
Objective response	11 (34.4)	20 (48.7)	
Disease control	21 (65.6)	26 (63.4)	

HCC, hepatocellular carcinoma; PVTT, portal vein tumor thrombosis; S-TACE, sorafenib-transarterial chemoembolization; A-TACE, apatinib-transarterial chemoembolization.

The tumor response was further analyzed according to the PVTT type (**Table 3**). The differences were not significant among patients with type I, II, and III of PVTT between S-TACE group and A-TACE group (*P* = 0.708, 0.632, and 1.000, respectively).

**Table 3:** PVTT response for patients with HCC and PVTT in the S-TACE or the A-TACE group.

Type	Response	Sorafenib + TACE group (n = 32) (cases %)	Apatinib + TACE group (n = 41) (cases %)	*P* value
I	Complete response	1 (3.1)	1 (2.4)	0.708
	Partial response	1 (3.1)	3 (7.3)	
	Stable disease	6 (18.8)	5 (12.2)	
	Progressive disease	2 (6.2)	4 (9.8)	
	Total	10 (31.3)	13 (31.7)	
II	Partial response	6 (18.8)	8 (19.5)	0.632
	Stable disease	9 (28.1)	12 (29.3)	
	Progressive disease	6 (18.8)	4 (9.8)	
	Total	21 (65.6)	24 (58.5)	
III	Partial response	0	1 (2.4)	1.000
	Stable disease	1 (3.1)	2 (4.9)	
	Progressive disease	0	1 (2.4)	
	Total	0	4 (9.8)	

HCC, hepatocellular carcinoma; PVTT, portal vein tumor thrombosis; S-TACE, sorafenib-transarterial chemoembolization; A-TACE, apatinib-transarterial chemoembolization.

**Figure 2:**
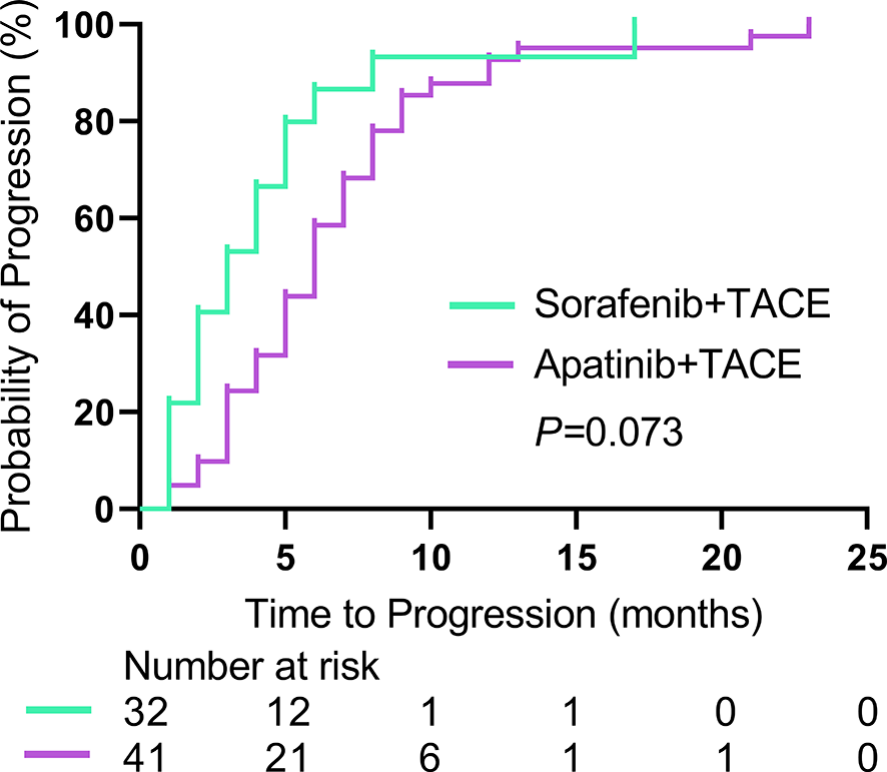
Kaplan-Meier curves show TTP in the sorafenib-TACE group and the apatinib-TACE group. TTP, time to progression; TACE, transarterial chemoembolization.

The median TTP was 5.0 months (95% CI: 2.9, 7.1) in the S-TACE group, and the median TTP was 6.0 months (95% CI: 4.2, 7.8) in the A-TACE group. The difference was not significant between the two groups (*P* = 0.073) (**Figure 2**).

**Figure 3:**
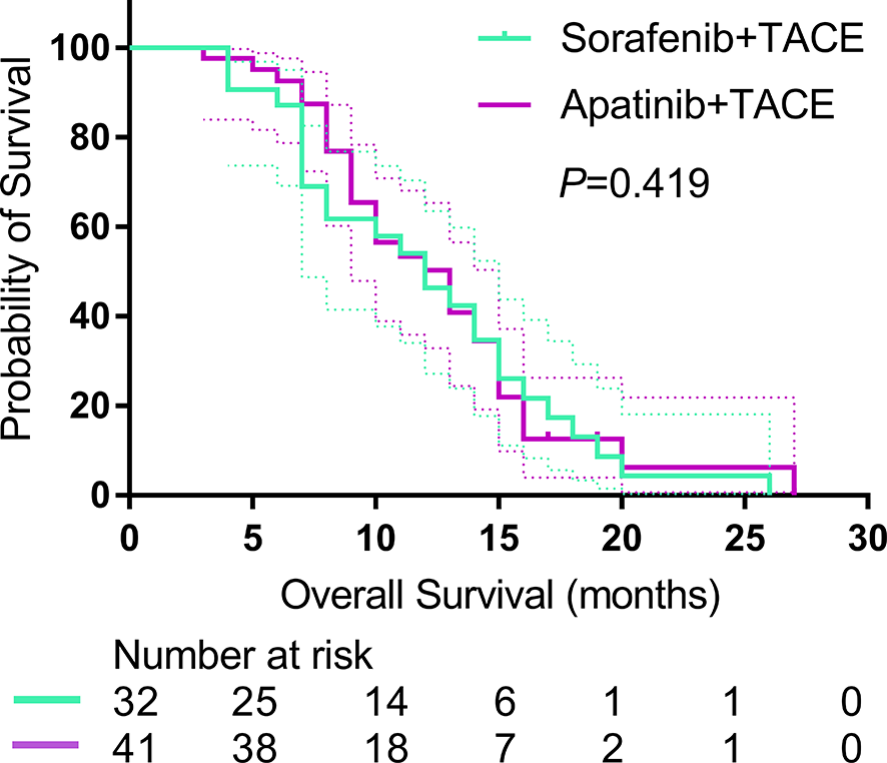
Kaplan-Meier curves show OS in the sorafenib-TACE group and the apatinib-TACE group. OS, overall survival; TACE, transarterial chemoembolization.

A total of 54 patients died at the end of the study (22 (81.2%) of 32 in the S-TACE group, and 32 (78.0%) of 41 in the A-TACE group). The median follow-up time was 10.0 months (range, 3.0–36.0 months) in the whole study. The median OS was 11.0 months (95% CI: 7.0, 15.0) and 10.0 months (95% CI: 9.4, 18.6) in the S-TACE and A-TACE group, respectively. The difference was not significant between the two groups (*P* = 0.419) (**Figure 3**).

### Subgroup Analysis

**Figure 4:**
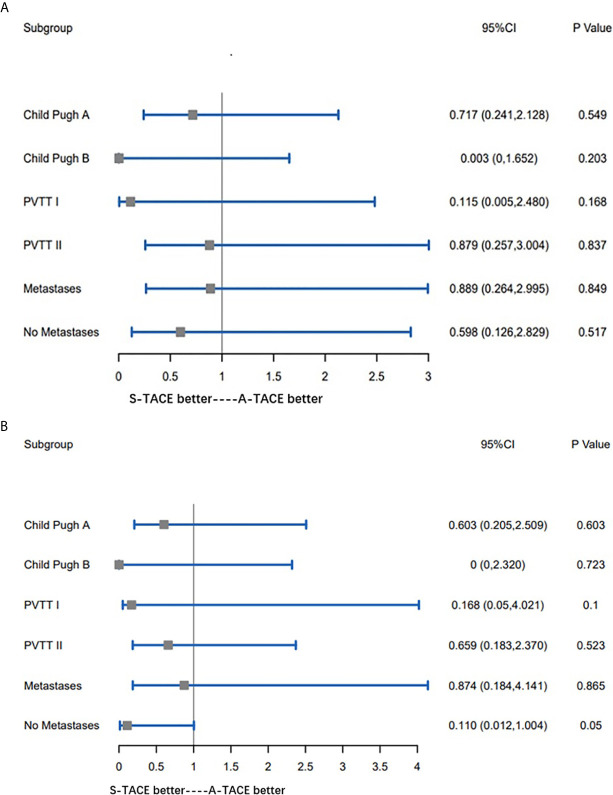
**(A)** Subgroup comparisons for OS of the prespecified subgroup metastasis (Yes or No), PVTT type (I and II), and Child-Pugh class **(A, B)**. **(B)** Subgroup comparisons for TTP of the prespecified subgroup metastasis (Yes or No), PVTT type (I and II), and Child-Pugh class **(A, B)**. OS, overall survival; PVTT, portal vein tumor thrombosis; TTP, time to progression.

Subgroup comparisons for OS of the prespecified subgroup metastasis (Yes or No), PVTT type (I and II), and Child-Pugh class (A and B). As shown in **Figure 4A**, there was no significant difference between any two groups. Similarly, subgroup analyses on TTP of the same prespecified factors also showed nonsignificant differences between any two groups (**Figure 4B**).

### Univariate and Multivariate Analysis

Prognostic factors of OS were presented in **Table 4**. Univariate analysis showed that Child-Pugh class (HR, 2.12; 95% CI, 1.22, 3.92), tumor diameter (HR, 1.84; 95% CI, 1.05, 3.23), and TACE number (HR, 0.58; 95% CI, 0.48, 0.71) were associated with OS. Further multivariate analysis indicated that the TACE number (HR, 0.61; 95%CI, 0.50, 0.75; *P* < 0.001) was an independent predictor of OS.

**Table 4:** Univariate and multivariate analysis of OS for patients with HCC and PVTT.

	Univariate analysis		Multivariate analysis	
	HR (95% CI)	P value	HR (95% CI)	P value
Age	1.12 (0.58, 2.17)	0.731		
≤60				
>60				
Gender	0.54 (0.21, 1.35)	0.185		
Male				
Female				
Bilirubin (μmol/l)	1.01 (0.99, 1.04)	0.251		
Albumin (g/l)	0.98 (0.93, 1.03)	0.389		
PT (s)	1.02 (0.85, 1.21)	0.859		
Child-Pugh class	2.12 (1.22, 3.92)	**0.009**	0.143 (0.77, 2.65)	0.262
A				
B				
AFP	0.883 (0.53, 1.48)	0.638		
<400				
>400				
ECOG	1.76 (0.94, 3.29)	0.079		
0				
1				
HBV infection	0.68 (0.34, 1.35)	0.273		
Yes				
No				
Number of tumors	0.93 (0.61, 1.40)	0.724		
≤3				
>3				
Tumor diameter	1.84 (1.05, 3.23)	**0.034**	1.37 (0.77, 2.44)	0.282
≤5				
>5				
PVTT type	0.93 (0.58, 1.47)	0.742		
1				
2				
3				
Ascites	1.42 (0.83, 2.44)	0.204		
Present				
Absent				
Extrahepatic spread	0.96 (0.57, 1.61)	0.862		
Present				
Absent				
TACE No.	0.58 (0.48, 0.71)	**<0.001**	0.61 (0.50, 0.75)	**<0.001**
Group	1.23 (0.73, 2.07)	0.442		
S-TACE				
A-TACE				

OS, overall survival; HCC, hepatocellular carcinoma; PVTT, portal vein tumor thrombosis; S-TACE, sorafenib-transarterial chemoembolization; A-TACE, apatinib-transarterial chemoembolization; AFP, a-fetoprotein; ECOG, Eastern Cooperative Oncology Group. The bold values means that the value of P < 0.05.

Prognostic factors of TTP were presented in **Table 5**. Univariate analysis showed that ECOG (HR, 1.85; 95% CI, 1.01, 3.39), tumor diameter (HR, 2.35; 95% CI, 1.37, 4.03), and TACE number (HR, 0.71; 95% CI, 0.60, 0.83) were associated with TTP. Further multivariate analysis indicated that the tumor diameter (HR, 1.84; 95% CI, 1.05, 3.23; *P* = 0.033) and TACE number (HR, 0.75; 95%CI, 0.64, 0.89; *P* = 0.001) were independent predictors of TTP in patients with PVTT.

**Table 5:** Univariate and multivariate analysis of TTP for patients with HCC and PVTT.

	Univariate analysis		Multivariate analysis	
	HR (95% CI)	P value	HR (95% CI)	P value
Age	1.44 (0.78, 2.65)	0.241		
≤60				
>60				
Gender	0.68 (0.32, 1.45)	0.318		
Male				
Female				
Bilirubin (μmol/l)	1.00 (0.98, 1.02)	0.814		
Albumin (g/l)	0.99 (0.95, 1.03)	0.593		
PT (s)	0.98 (0.85, 1.12)	0.724		
Child-Pugh class	1.60 (0.92, 2.78)	0.095		
A				
B				
AFP	0.98 (0.61, 1.57)	0.923		
<400				
>400				
ECOG	1.85 (1.01, 3.39)	**0.046**	1.34 (0.71, 2.52)	0.368
0				
1				
HBV infection	0.86 (0.46, 1.60)	0.626		
Yes				
No				
Number of tumors	1.10 (0.77, 1.55)	0.608		
≤3				
>3				
Tumor diameter	2.35 (1.37, 4.03)	**0.002**	1.84 (1.05, 3.23)	**0.033**
≤5				
>5				
PVTT type	1.02 (0.67, 1.55)	0.933		
1				
2				
3				
Ascites	1.44 (0.87, 2.40)	0.159		
Present				
Absent				
Extrahepatic spread	0.85 (0.53, 1.38)	0.516		
Present				
Absent				
TACE No.	0.71 (0.60, 0.83)	**<0.001**	0.75 (0.64, 0.89)	**0.001**
Group	0.67 (0.42, 1.09)	0.107		
S-TACE				
A-TACE				

TTP, time to progression; HCC, hepatocellular carcinoma; PVTT, portal vein tumor thrombosis; S-TACE, sorafenib-transarterial chemoembolization; A-TACE, apatinib-transarterial chemoembolization; AFP, a-fetoprotein; ECOG, Eastern Cooperative Oncology Group. The bold values means that the value of P < 0.05.

### Safety Assessment

In addition to postembolization syndrome, no severe adverse events that are related to TACE procedure were observed. The adverse events that are related to sorafenib or apatinib were listed in **Table 6**. The most frequent adverse reactions in both groups were similar, which included hand-foot skin reactions, diarrhea, hypertension, proteinuria, and fatigue. Most patients in the two groups suffered grade 1–3 adverse events, and three patients underwent grade 4–5 reactions in both groups. All the patients with adverse events were alleviated or eliminated after drug reduction or with symptomatic treatments. No treatment-related fatal adverse events were observed. The liver function was not significantly different between the two groups, and the changes were comparable to TACE monotherapy according to our clinical observation (data not shown).

**Table 6:** Adverse events related to sorafenib or apatinib in the S-TACE or the A-TACE group.

Adverse events	S-TACE group (n = 32) (cases %)		A-TACE group (n = 41) (cases %)	
	Grade 1–3	Grade 4–5	Grade 1–3	Grade 4–5
Hand-foot skin reactions	24 (75)	1 (3.1)	30 (73.2)	1 (2.4)
Hypertension	11 (34.4)	1 (3.1)	18 (43.9)	1 (2.4)
Diarrhea	23 (71.9)	0	27 (65.9)	0
Fatigue	9 (28.1)	0	10 (24.4)	0
Headache	5 (15.6)	0	5 (12.2)	0
Oral ulcer	3 (9.4)	0	4 (9.8)	0
Voice change	0	0	2 (4.9)	0
Proteinuria	10 (31.3)	0	11 (26.8)	0
Gastrointestinal hemorrhage	1 (3.1)	1 (3.1)	2 (4.9)	1 (2.4)
New hypothyroidism	1 (3.1)	0	1 (2.4)	0

S-TACE, sorafenib-transarterial chemoembolization; A-TACE, apatinib-transarterial chemoembolization. The bold values means that the value of P < 0.05.

## Discussion

Existing evidences for the efficacy of S-TACE or A-TACE on advanced HCC with PVTT all focus on the outcome comparison between the combination therapy and the monotherapy (15, 16, 21, 22). The present study for the first time directly compared the combination prognosis between S-TACE and A-TACE for HCC patients with PVTT.

Our results demonstrated similar prognosis of HCC patients with PVTT who were treated with either S-TACE or A-TACE. In addition, the OS in the S-TACE group was 11.0 months, which was comparable to previous researches of 9–14 months (4, 25). The OS in the A-TACE group was 10.0 months, which was slightly inferior to Liu et al. of 11.9 months (22), although it could not be compared directly because the two studies were different. Since some researches revealed that the prognosis was different for HCC patients with different type and response of PVTT (3, 26), we further compared the response of PVTT according to their type, and the OS according to the response of PVTT (data not shown). However, the differences were still not significant. Besides, the TTP and the tumor response between the two groups were also not significantly different.

There is synergistic effect of combining TACE with molecular targeted drugs, such as sorafenib and apatinib. Local-reginal therapeutic efficacy of tumor burden can be reduced by TACE. Sorafenib and apatinib inhibit the elevated VEGF level after TACE procedure and, more importantly, play a systemic therapeutic role. Therapeutic mechanisms of sorafenib and apatinib are not exactly the same, but our results indicated that the synergistic effect of the two tyrosine-kinase inhibitors with TACE was similar. In contrast, other multitargeted molecular targeting agents, such as sunitinib and linifanib, could not benefit HCC patients as first-line therapy when compared with sorafenib. In theory, apatinib may exert a stronger anti-angiogenesis effect than sorafenib due to its 10 times higher binding affinity with VEGFR-2. Thus, the hypothesis could not be excluded that when compared with sorafenib, the potent VEGFR-2 inhibition ability of apatinib compensates its fewer inhibition targets when combined with TACE in HCC patients with PVTT. However, the presume needs to be further identified.

The multivariate analysis revealed that the PVTT type and extrahepatic metastasis were not prognostic factors for OS, indicating the comparable inhibitory effect of S-TACE and A-TACE on the metastasis of HCC to some extent. Besides, the number of TACE was a protective factor for OS and TTP, illustrating the importance of TACE in the two combination treatments. Although the possibility that the longer survival provided more chances for performing more TACE treatments could not be excluded, researches demonstrated that the repeat TACE improves the prognosis of HCC patients. For example, Duan et al. have shown that the repeat TACE treatment for patients with residual or recurrent HCC can achieve substantial response rates of over 50% (27). Moreover, Georgiades et al. and Chen et al. demonstrated that the repeat TACE procedure can obtain better regional responses and OS even for those who were previously non-responders to TACE (28, 29).

Taken together, these results revealed that A-TACE with a lower medical cost could be an effective alternate of S-TACE for HCC patients with PVTT, especially for those who could not cover the cost of sorafenib. The conclusion would be more convincing if large-scale randomized controlled trials are conducted in the future.

The BCLC guidelines suggested that TACE is not suitable for HCC patients with PVTT because it may further worsen the previously poor liver function supply and liver function caused by PVTT. However, emerging studies have demonstrated that TACE is a safe and effective method in selected HCC patients with good hepatic function and established collateral blood circulation around the occluded portal vein (26). The present study reached a similar conclusion, as the liver function have not been impaired additionally after S-TACE or A-TACE, and there were no treatment-related death and few grade 4–5 adverse events that occurred. Besides, the most frequent adverse events related to sorafenib and apatinib in both groups were similar, including hand-foot skin reactions, diarrhea, hypertension, proteinuria, and fatigue, which were consistent with previously reported ones in the monotherapy of sorafenib and apatinib (30). These results indicated that the two combination treatments were tolerable to HCC patients with PVTT.

There were several limitations in the study. First, this was a retrospective, single-center study, and the sample size was relatively small. Second, the more convincing propensity score matching analyses have not been performed, although the differences of baseline characteristics between groups were comparable. Third, CT-scan has limitations in evaluating the viable tumor tissues after lipiodol staining, which was further confirmed by MRI or DSA if two experienced imaging experts could not reach a consensus. Fourth, the decision to treat with sorafenib or apatinib was made by the consensus of the treating physicians and their patients; thus, there might be selection bias.

## Conclusion

S-TACE and A-TACE exhibited comparable tumor response, TTP, and OS in advanced HCC patients with PVTT, which provide another effective method for A-TACE in addition to conventional treatment of S-TACE for these patients, especially for those who have medical concerns about the high price of sorafenib.

## Data Availability Statement

The raw data supporting the conclusions of this article will be made available by the authors, without undue reservation.

## Ethics Statement

The studies involving human participants were reviewed and approved by the Ethics Committee of Tongji Medical college, Huazhong University of Science and Technology. Written informed consent for participation was not required for this study in accordance with the national legislation and the institutional requirements.

## Author Contributions

Conceptualization, YC, TS, MW, and CZ. Methodology, YC. Validation, TS, LC, and CZ. Formal analysis, YC, LC, XK, and BL. Writing—original draft preparation, YC. Writing—review and editing, TO. Visualization, XG. Supervision, CZ and MW. All authors contributed to the article and approved the submitted version.

## Conflict of Interest

The authors declare that the research was conducted in the absence of any commercial or financial relationships that could be construed as a potential conflict of interest.

## Publisher’s Note

All claims expressed in this article are solely those of the authors and do not necessarily represent those of their affiliated organizations, or those of the publisher, the editors and the reviewers. Any product that may be evaluated in this article, or claim that may be made by its manufacturer, is not guaranteed or endorsed by the publisher.
